# Association Between Prescribed Dosage of Resistance Exercise and Change in Pain and Physical Function in Knee Osteoarthritis: A Systematic Review With Meta‐Regression

**DOI:** 10.1002/msc.70110

**Published:** 2025-05-04

**Authors:** Jelke de Wit, Travis Haber, Michelle Hall, Kim L. Bennell, Rana S. Hinman, Libby Spiers, Alexander J. Kimp, Andrea Dell’Isola, Alison R. Harmer, Martin van der Esch, Belinda J. Lawford

**Affiliations:** ^1^ Radboud University Nijmegen the Netherlands; ^2^ The University of Melbourne Victoria Australia; ^3^ The University of Sydney Sydney Australia; ^4^ Lund University Lund Sweden; ^5^ Amsterdam University of Applied Sciences Amsterdam the Netherlands

**Keywords:** dosage, exercise, knee, osteoarthritis, pain, rehabilitation, systematic review

## Abstract

**Objective:**

To assess whether there is an association between total prescribed dosage of resistance exercise (volume, frequency, intensity, and duration) and change in pain and physical function in individuals with knee osteoarthritis (OA).

**Method:**

A systematic review with meta‐regression was conducted, searching MEDLINE, Embase, and the Cochrane Central Register of Controlled Trials until December 11, 2024. We included randomised controlled trials that compared resistance exercise for knee OA with non‐exercise interventions. Meta‐regression examined the association between total prescribed exercise dosage (volume × frequency × intensity × duration of the intervention) and standardised mean differences (SMDs) for change in pain and physical function. Covariates were included to attempt to reduce between‐study heterogeneity.

**Results:**

Analysis of 14 trials (*N* = 1274) found no association between total prescribed resistance exercise dosage and changes in pain (slope coefficient: < 0.01 on a 0–100 scale [95% CI: < − 0.01 to < 0.01]; *p* = 0.18) or physical function (slope coefficient: < 0.01 on a 0–100 scale [95% CI: < − 0.01 to < 0.01]; *p* = 0.15). Heterogeneity was substantial (*I* ^2^ = 73%–97%) and many trials were of unclear/high risk of bias.

**Conclusion:**

No association was found between the total prescribed dosage of resistance exercise and changes in pain or function in individuals with knee OA. However, due to the limited number of trials, high heterogeneity, and overall low quality of studies, findings should be interpreted with caution.

## 1 Background

Osteoarthritis (OA) affects approximately 654 million adults worldwide (Cui et al. 2020), causing chronic pain and affecting daily activities and well‐being (Lai et al. 2021; Wallis et al. 2019). Clinical guidelines advocate exercise for managing knee OA, irrespective of age, comorbidity, pain severity, or disability (Bannuru et al. 2019; Kolasinski et al. 2020; Moseng et al. 2024; National Institute for Health and Care Excellence, 2022; The Royal Australian College of General Practitioners, 2018). Although numerous systematic reviews support the effectiveness of exercise, effects are of uncertain clinical importance and decline over time (Holden et al. 2023; Lawford et al. 2024). New ways of enhancing the effectiveness of exercise are needed, such as by identifying the optimal dosage of exercise programs.

Given muscle weakness is common in people with OA (Hinman et al. 2010; Muraki et al. 2015; Vårbakken et al. 2019), and increasing muscle strength is hypothesised to be one mechanism by which resistance exercise could lead to improvements in symptoms (Bandak et al. 2019; Hall et al. 2018; Muraki et al. 2015; Runhaar et al. 2023; Runhaar et al. 2015; Vårbakken et al. 2019), resistance exercise is commonly used for management of OA. Some research in adults without OA suggests there is a dose‐response relationship between resistance exercise and strength gains (Peterson et al. 2005; Rhea et al. 2003). As such, it could be assumed that, in OA, a higher dosage (i.e., higher volume, frequency, and/or intensity) may result in greater improvements in symptoms. However, recent studies suggest that this may not be the case (de Zwart et al. 2022; Husted et al. 2022; Messier et al. 2021; Torstensen et al. 2023; Turner et al. 2020). The optimal resistance exercise dosage for knee OA symptoms has not yet been established (Moseng et al. 2024).

A recent systematic review aimed to investigate dosage parameters of resistance exercise for people with knee and hip OA (Marriott et al. 2024). They found that effects on symptoms were not associated with exercise volume (defined as frequency × session duration × length of the intervention). However, that review included trials with any kind of comparator (including active interventions like other types of exercise), and thus their findings may not represent the isolated effects of resistance exercise alone on symptoms (Marriott et al. 2024). That review also did not consider the effects of other key dosage parameters such as intensity, repetitions, sets, or the number of exercises, and thus the influence of these other important variables on outcomes remains unclear (Holden et al. 2021).

Another systematic review aimed to determine whether there is a resistance exercise dose‐response relationship in people with knee OA (Wang et al. 2024). In a series of individual meta‐regressions, the authors analysed effects of various dosage variables, including length of the intervention, intensity, frequency, sets, and repetitions. They found that only length of the intervention was associated with pain and function outcomes, though did not analyse whether total dosage (taking into account the combined effects of all relevant exercise variables together) related to treatment effects, and also did not consider number of different exercises prescribed (Wang et al. 2024).

This study aimed to explore the association between total prescribed resistance exercise dosage (taking into account intensity, frequency, sets, repetitions, number of exercises, and duration of the intervention) and changes in pain and physical function in adults with knee OA. As a secondary objective, this study aimed to determine whether any of those individual exercise dosage variables are associated with changes in pain and physical function.

## 2 Methods

This systematic review adhered to the Cochrane Handbook for Systematic Reviews of Interventions (Higgins, 2008), and was pre‐registered in the International Prospective Register of Systematic Reviews (PROSPERO CRD42025631018). It was reported according to the guidelines outlined in the Preferred Reporting Items for Systematic Reviews and Meta‐Analyses (PRISMA) statement (Page et al. 2021).

### 2.1 Literature Search

We used data extracted as part of a Cochrane Systematic Review update evaluating the effectiveness of exercise for knee OA (Lawford et al. 2024). We also conducted a search update in the Cochrane Central Register of Controlled Trials (CENTRAL), Embase, and MEDLINE from January 4, 2024 (the date of the last search for the Cochrane review) to December 11, 2024. Search results were managed using Covidence. Grey literature was not included. The search strategy is detailed in Appendix 1.

### 2.2 Study Selection Criteria

We included RCTs that aligned with our specified population, intervention, and comparator groups. There were no language restrictions.

Trials were eligible if they involved adults with:

- Knee OA diagnosed in accordance with accepted clinical and/or radiographic criteria (Altman, 1991; National Institute for Health and Care Excellence, 2022; Zhang et al. 2010); or
- Self‐reported knee OA based on chronic joint pain, with or without radiographic confirmation; and
- OA in other joints, provided that the results for participants with knee OA were reported separately (or could be provided by the authors), or if knee OA was present in at least 80% of the participants.

We included any RCT that involved a land‐based resistance exercise programme, that is, programs that used voluntary muscle contractions against resistance, such as machines, resistance bands, body weight, or free weights, as defined in the American College of Sports Medicine guidelines (Liguori & Medicine, 2020).

Exercise interventions eligible for inclusion:

- May include other types of exercise (e.g., stretching), but only during warm‐up or cool‐down periods.
- May allow participants to receive educational or behaviour‐change strategies designed to improve adherence to the exercise programme.
- May be either supervised or unsupervised.
- May incorporate non‐surgical co‐interventions (e.g., injections), provided these were applied similarly in the comparator group.

We permitted the inclusion of 3‐arm trials employing the same type and dosage of resistance exercise in each exercise group (e.g., Group A: resistance training conducted in individual sessions; Group B: the identical resistance programme conducted in group sessions; Group C: control).

Exercise interventions were ineligible if they:

- Involved whole‐body vibration or gait retraining.
- Included any exercise other than resistance exercise (e.g., balance training, stretching, or mind‐body exercises such as yoga or Tai Chi).
- Were perioperative (involving participants post‐surgery or on a surgical waiting list).
- Did not report all prescribed dosage variables of interest (i.e., volume [sets, repetitions, and number of exercises], frequency per week, intensity, and duration of the intervention).

Comparator groups eligible for inclusion were:

- Placebo, sham, or attention control groups (i.e., interventions intended to control for placebo or contextual effects, referred to as ‘placebo/sham’, and/or ‘attention control’ interventions involving at least one instance of direct interaction with a care provider, excluding communication with study staff solely for the purpose of collecting outcome data).
- No treatment, standard care, or limited education (i.e., participants were given a single informational resource).
- Any non‐exercise, non‐surgical intervention that was offered or provided equally as a co‐intervention in the exercise group (e.g., weight loss diet, manual therapy, physical therapy [excluding any exercise component]).

Trials were considered eligible if they included at least one of the following outcomes;

- Self‐reported knee pain;
- Self‐reported physical function.

### 2.3 Study Selection

Teams of two review authors independently screened titles/abstracts and full‐texts in Covidence to identify all potentially relevant studies. If a study was ineligible, the reason for exclusion was provided. Any conflicts were resolved through group discussion.

### 2.4 Quality Assessment

For consistency with the overarching Cochrane review (Lawford et al. 2024), we used Tool 1 from the Cochrane Collaboration's Risk of Bias to assess potential biases in included RCTs (Higgins JPT et al. 2022). Each RCT was independently evaluated for risk of bias by two authors, with each bias domain rated as ‘low risk’ (adequate), ‘high risk’ (inadequate), or ‘unclear’ (insufficient information). Bias domains included random sequence generation and allocation concealment (selection bias), blinding of participants and personnel (performance bias), blinding of outcome assessment (detection bias), incomplete outcome data (attrition bias), and selective reporting of outcomes (reporting bias). Any conflicts were resolved through group discussion.

### 2.5 Data Collection

Data were independently extracted from included studies by two review authors using a customised form in Covidence. This included information about study participants (number randomised to each group, mean age, percentage of females, body mass index, and diagnostic criteria for OA), prescribed exercise interventions (number of strengthening exercises, number of sets and repetitions, intensity, number of sessions per week, duration of the intervention, number of clinician consultations), and type of comparator. We extracted the prescribed dosage rather than the dosage actually performed, as actual dosage performed is often unmeasured and/or not reported in clinical trials.

Means and standard deviations (SD) of outcomes (i.e., pain and function) were extracted immediately after the treatment phase (post‐treatment). In cases where multiple pain or function measures were reported, a hierarchy of outcomes was applied, as outlined in the overarching Cochrane review (Lawford et al. 2024). If both post‐treatment scores and changes from baseline were provided, post‐treatment values were extracted. For missing outcome data, efforts were made to contact the trial authors.

### 2.6 Data Analysis

We calculated standardised mean differences (SMDs) with 95% confidence intervals (CI) and standard error (SE) for both pain and function. We ensured data were entered using a consistent direction of effect across all studies. To aid interpretation, SMDs were back‐transformed to a 0–100 scale using the weighted baseline SD of comparison groups. In multi‐arm trials, only relevant arms were included. If trials included more than one exercise group, and exercise groups had the same dosage, groups were pooled to compute the SMD.

For each trial, we calculated a unitless variable representing total dosage of the exercise programme using the following formula: (volume [number of sets × number of repetitions × number of different exercises] × frequency per week × intensity × duration of the intervention) (Lyristakis et al. 2022; Young et al. 2018).

Intensity was converted to a consistent scale (0–10 rating of perceived exertion [RPE]) using established methods of conversion (Morishita et al. 2018; Reynolds et al. 2006; Row et al. 2012). If different resistance exercise intensity parameters were prescribed within a single session or throughout the intervention period, the average was calculated and rounded to one decimal place.

We conducted meta‐regressions to examine the association between total resistance exercise dosage and change in pain and physical function. The *I* ^2^ statistic was used to assess study heterogeneity. We used random‐effects analyses, anticipating heterogeneity in SMDs due to variations in study characteristics. Two pre‐specified study‐level covariates were included to attempt to address between‐study heterogeneity: (1) type of comparator (attention control/placebo, no treatment/usual care/limited education, or a co‐intervention that was also equally applied in the exercise group), and (2) the total number of consultations with a clinician throughout the intervention. These covariates were selected based on their theoretical plausibility (determined via discussions with review authors with relevant content expertise) and evidence from previous exercise meta‐regressions (Juhl et al. 2014; Marriott et al. 2024). Meta‐regressions were also conducted without the inclusion of any covariates. We conducted Egger's regression test and examined the funnel plots to assess potential publication bias (Sterne & Egger, 2005). We performed sensitivity analyses to determine whether results differed when excluding low‐quality trials, defined as those with an unclear or high risk of bias in ≥ 3 out of six bias domains.

We performed secondary exploratory analyses to further investigate associations between each individual dosage variable and outcomes. This involved conducting additional meta‐regressions where the four main individual dosage variables contributing to the above calculation (i.e., volume [sets × repetitions × number of exercises], frequency per week, intensity, duration) were added to the model separately, rather than as one total dosage variable. This was done including the covariates (i.e., type of comparator group and number of consultations with a clinician).

## 3 Results

### 3.1 Study Selection

**FIGURE 1:**
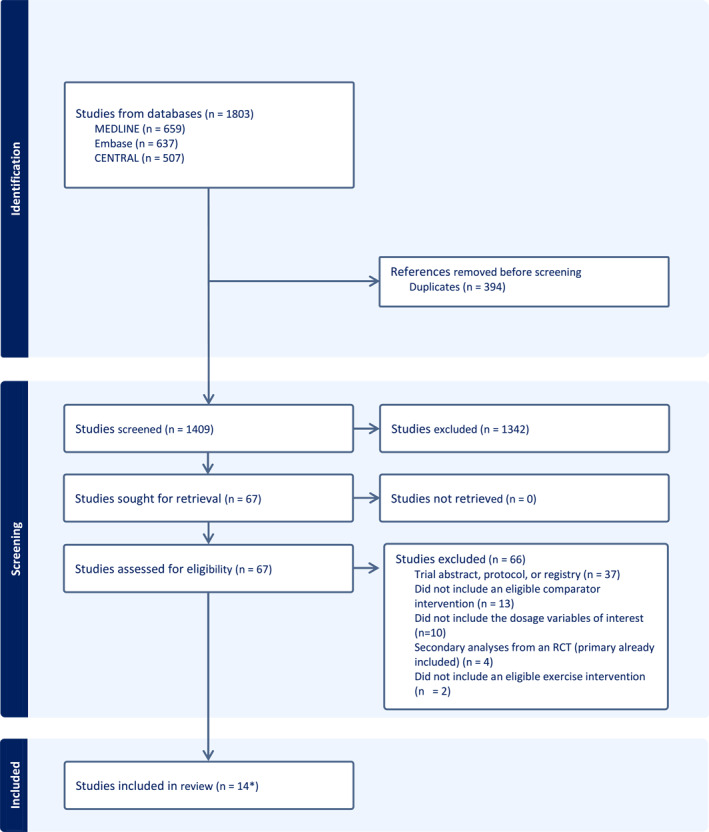
Flowchart of studies included in the systematic review and meta‐analysis. * Including an additional 13 trials from the prior version of the Cochrane review (Lawford et al. 2024).

The initial search identified 1803 articles, with 1409 remaining after removing duplicates and 67 remaining for full‐text review. After full‐text review, 14 studies, with 1274 participants, were included in the analyses (Figure 1).

### 3.2 Study Characteristics

Study characteristics are shown in Appendix 2. Four trials (29%) compared resistance exercise with placebo or attention control (Baker et al. 2001; de Almeida et al. 2020; Ettinger et al. 1997; Imoto et al. 2012). Five trials (36%) compared resistance exercises with no intervention or usual care (Bennell et al. 2010; Bruce‐Brand et al. 2012; Lee et al. 2023; Pazit et al. 2018; Topp et al. 2002), and five trials (36%) compared resistance exercises with a co‐intervention that was equally applied in the exercise group (Chang et al. 2012; Hsu et al. 2021; Jan et al. 2009; Karaborklu Argut et al. 2024; Samuel Sundar Doss et al. 2014).

The average age of participants was 62.9 years (SD 6.0; range 50.4–69.1 years), with a mean BMI of 28.7 (SD 2.6 kg/m^2^; range 24.9–32.8 kg/m^2^). The percentage of women in each study varied from 48% to 100% (mean 73%; SD 16%). The median total resistance exercise dosage of exercise in the intervention group was 17,520 (interquartile range 5180–29,808).

Pain was measured in all but one study (Jan et al. 2009). Seven studies (50%) used the Western Ontario and McMaster Universities Arthritis Index (WOMAC) or the Knee Injury and Osteoarthritis Outcome Score (KOOS) (Baker et al. 2001; Bruce‐Brand et al. 2012; Chang et al. 2012; Hsu et al. 2021; Pazit et al. 2018; Samuel Sundar Doss et al. 2014; Topp et al. 2002). Five studies (36%) used a Numeric Rating Scale (NRS) or Visual Analogue Scale (VAS) (Bennell et al. 2010; de Almeida et al. 2020; Imoto et al. 2012; Karaborklu Argut et al. 2024; Lee et al. 2023). One study (7%) used a Likert scale (Ettinger et al. 1997).

Function was measured in all but one study (Lee et al. 2023). The WOMAC function subscale or KOOS Activities of Daily Living was used in 11 studies (79%) (Baker et al. 2001; Bennell et al. 2010; Bruce‐Brand et al. 2012; Chang et al. 2012; de Almeida et al. 2020; Hsu et al. 2021; Jan et al., 2009; Karaborklu Argut et al. 2024; Pazit et al. 2018; Samuel Sundar Doss et al. 2014; Topp et al. 2002). One study (7%) used a 1 to 6 scale (Ettinger et al. 1997) and one study (7%) used the Short Form Survey (SF‐36) (Imoto et al. 2012).

### 3.3 Risk of Bias

**FIGURE 2:**
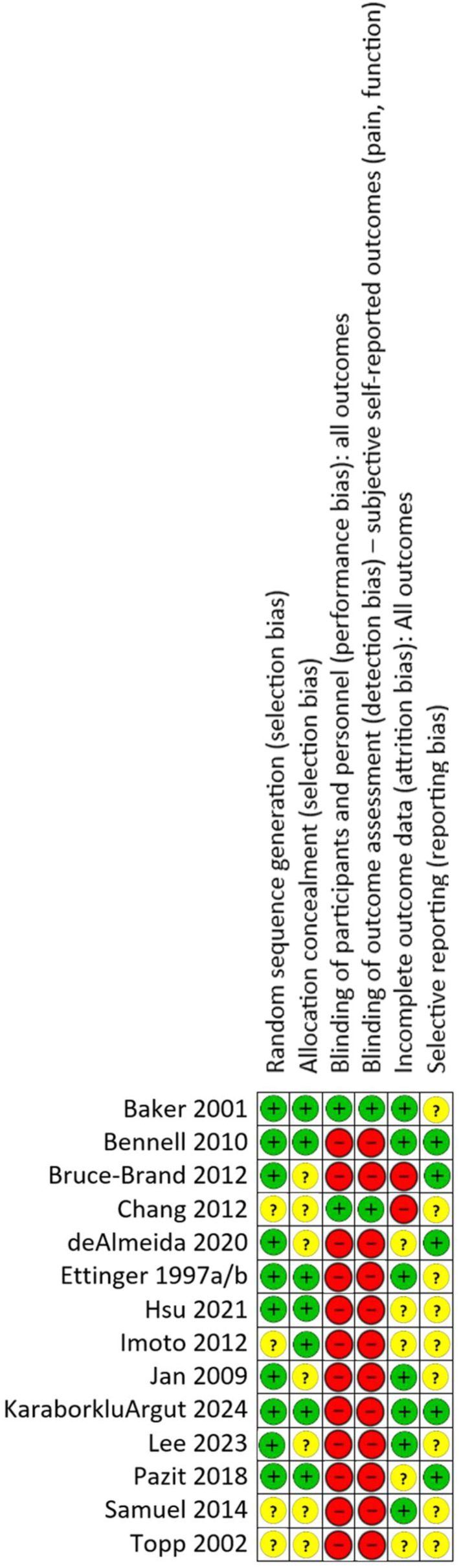
Risk of bias of included studies.

Figure 2 presents the risk of bias of the included trials. Five studies (36%) had low overall risk of bias (i.e., low risk of bias in four or more domains) (Baker et al. 2001; Bennell et al. 2010; Ettinger et al. 1997; Karaborklu Argut et al. 2024; Pazit et al. 2018). Six studies (43%) were considered to be at low risk of selection bias (Baker et al. 2001; Bennell et al. 2010; Ettinger et al. 1997; Hsu et al. 2021; Karaborklu Argut et al. 2024; Pazit et al. 2018). Two studies (14%) blinded participants through limited disclosure (Baker et al. 2001; Chang et al. 2012), and were thus considered to be at low risk of both performance and detection bias. Seven studies (50%) were considered to be at low risk of attrition bias (Baker et al. 2001; Bennell et al. 2010; Ettinger et al. 1997; Jan et al., 2009; Karaborklu Argut et al. 2024; Lee et al. 2023; Samuel Sundar Doss et al. 2014). Five studies (36%) were considered to be at low risk of reporting bias (Bennell et al. 2010; Bruce‐Brand et al. 2012; de Almeida et al. 2020; Karaborklu Argut et al. 2024; Pazit et al. 2018).

### 3.4 Association Between Total Prescribed Exercise Dosage and Pain

**FIGURE 3:**
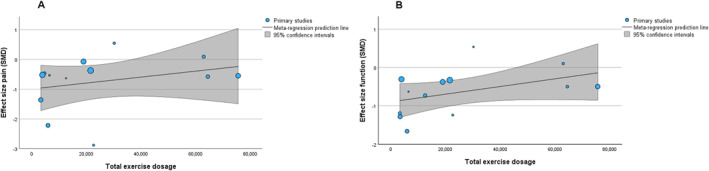
Meta‐regression analysis. The SMD (standardised mean difference) of the included studies for pain (A) and physical function (B) in relation to total exercise dosage. Lower SMD values indicate a favourable effect of exercise. The analysis accounted for the following covariates: (1) type of comparator (attention control/placebo, no treatment/usual care/limited education, or a co‐intervention equally applied in the exercise group), and (2) the total number of consultations with a clinician throughout the intervention.

Meta‐regressions showed no association between total dosage of prescribed exercise and change in pain (slope coefficient: < 0.01 on a 0–100 scale [95% CI: < − 0.01 to < 0.01]; *p* = 0.18; Figure 3A). Substantial heterogeneity (*I* ^2^ = 97%) was observed. In an unadjusted model without covariates, there was also no association between total dosage and change in pain (Appendix 3). No association was found between change in pain and individual dosage variables, including frequency, volume, intensity, or duration (Appendix 4). Egger's test and funnel plot indicated no risk of publication bias (Appendix 5). A sensitivity analysis including only studies of low risk of bias could not be conducted due to an insufficient number of studies.

### 3.5 Association Between Total Prescribed Exercise Dosage and Function

Meta‐regression estimates showed no association between the total prescribed dosage of exercise and change in function (slope coefficient: < 0.01 on a 0–100 scale [95% CI: < − 0.01 to < 0.01]; *p* = 0.15; Figure 3B). Substantial heterogeneity (*I* ^2^ = 73%) was observed. In an unadjusted model without any covariates, there was also no association between total dosage and change in function (Appendix 3). However, there was an association between volume and change in function (Appendices 4 and 6), whereby a higher volume was associated with reduced effect on function. Specifically, a 1‐unit increase in volume was associated with a 0.09‐unit reduced improvement in physical function on a 0–100 scale (95% CI: < 0.01–0.15]; *p* = 0.049). There was no association between change in function and other individual dosage variables, including frequency, intensity, or duration (Appendix 4). Egger's test and funnel plot indicated no risk of publication bias (Appendix 5). A sensitivity analysis including only studies of low risk of bias could not be conducted due to the insufficient number of these studies.

## 4 Discussion

The aim of this systematic review was to examine whether there is an association between the total dosage of prescribed resistance exercise and change in pain or function in people with knee OA. We found no association, suggesting that total dosage may not be an important factor when prescribing resistance exercises to treat knee OA. However, given the limited number of included trials, overall poor quality of the included studies, and the high heterogeneity, these findings should be interpreted with caution.

To our knowledge, this is the first systematic review to assess the association between the total prescribed dosage of resistance exercise (i.e., volume, frequency, intensity, and duration) with pain and function in people with knee OA. Our findings align with previous research. Another systematic review (Marriott et al. 2024), including 151 knee OA studies, found no association between prescribed exercise volume (defined as frequency, time, and duration) and outcomes. Our findings also align with various RCTs (de Zwart et al. 2022; Husted et al. 2022; Messier et al. 2021; Torstensen et al. 2023), which found no differences in pain and function when comparing low volume/intensity/frequency/duration with high volume/intensity/frequency/duration. Collectively, this suggests that an association between prescribed exercise dosage and exercise effects on pain and physical function may not exist in knee OA. Furthermore, current evidence suggests that the mechanisms by which exercise influences pain and physical function remain uncertain (Henriksen et al. 2024), and increases in muscle strength may contribute only a small fraction of the overall improvements in pain and physical function in OA (Runhaar et al. 2023). Psychological factors (e.g., pain beliefs, fear of movement, and self‐efficacy), as well as contextual factors and placebo responses, are likely to play an important role (Bandak et al. 2022; Henriksen et al. 2023; Ribeiro et al. 2024; Zhang et al. 2008).

Our findings have implications for healthcare providers. Given that our findings suggest no association between the total dosage of resistance exercise and pain or function, healthcare providers may consider using a more individualised dosage prescription rather than assuming a greater total prescribed dosage of exercise will optimise clinical outcomes. Interestingly, our exploratory meta‐regression identified a potential negative association between exercise volume and function. However, this finding should be interpreted with caution and further research, including high‐quality clinical trials, is needed to explore this potential relationship.

We have low certainty in our review findings. Although > 65 RCTs were potentially eligible for inclusion (i.e., involved resistance exercise and had an eligible comparator), only 14 of these reported all prescribed dosage parameters and could be included in our analysis. There was also substantial between‐study heterogeneity, even after adjusting for covariates. Additionally, 64% of included studies had a high or unclear overall risk of bias, indicating poor methodological quality. Future trials should minimise bias by improving allocation concealment, using intention‐to‐treat analyses, and prospectively registering in a trial registry. There is also a clear need for better reporting on prescription of exercise interventions (Hansford et al. 2022), including reporting of key dosage variables such as repetitions, sets, number of exercises, frequency, and intensity. Trial authors should adhere to established reporting guidelines, including the Consolidated Standards of Reporting Trials (CONSORT) (Moher et al. 2010) checklist when presenting results, as well as the Consensus on Exercise Reporting Template (CERT) (Slade et al. 2016) and the Template for Intervention Description and Replication (TIDieR) (Hoffmann et al. 2014) checklist when detailing exercise protocols.

Our systematic review has limitations. Only 14 trials were eligible for inclusion, which may impact the reliability and generalisability of the findings. Additionally, our analyses were based on the prescribed exercise dosage, rather than the actual exercise dosage performed by participants. Participant adherence to the exercise programme was not taken into account, as it was inconsistently reported, which could have influenced outcomes. It is also possible that the total dosage of resistance exercises is associated with outcomes that were not assessed in our study (e.g., muscle strength). Finally, as our review focused specifically on resistance exercise and knee OA, our findings may not reflect potential associations between exercise dosage and outcomes in other types of exercise or in OA in other joints, such as the hip.

## 5 Conclusion

No association was found between the total prescribed dosage of resistance exercise and changes in pain or function in individuals with knee OA. However, due to the limited number of trials, high heterogeneity, and overall low quality of studies, findings should be interpreted with caution.

## Author Contributions

**Jelke de Wit:** conception and design, draughting of the article, critical revision of the article for important intellectual content, final approval of the article, provision of study materials or patients, collection and assembly of data. **Travis Haber:** conception and design, draughting of the article, critical revision of the article for important intellectual content, final approval of the article, provision of study materials or patients, collection and assembly of data, statistical expertise. **Michelle Hall:** critical revision of the article for important intellectual content, final approval of the article, provision of study materials or patients, collection and assembly of data. **Kim L. Bennell:** critical revision of the article for important intellectual content, final approval of the article, provision of study materials or patients, collection and assembly of data. **Rana S. Hinman:** critical revision of the article for important intellectual content, final approval of the article, provision of study materials or patients, collection and assembly of data. **Libby Spiers:** critical revision of the article for important intellectual content, final approval of the article, provision of study materials or patients, collection and assembly of data. **Alexander J. Kimp:** critical revision of the article for important intellectual content, final approval of the article, provision of study materials or patients, collection and assembly of data. **Andrea Dell’Isola:** critical revision of the article for important intellectual content, final approval of the article, provision of study materials or patients, collection and assembly of data. **Alison R. Harmer:** critical revision of the article for important intellectual content, final approval of the article, provision of study materials or patients, collection and assembly of data. **Martin van der Esch:** critical revision of the article for important intellectual content, final approval of the article, provision of study materials or patients, collection and assembly of data. **Belinda J. Lawford:** conception and design, draughting of the article, critical revision of the article for important intellectual content, final approval of the article, provision of study materials or patients, collection and assembly of data

## Conflicts of Interest

The authors declare no conflicts of interest.

## Data Availability

The data that support the findings of this study are available from the corresponding author upon reasonable request.

## Appendix 1

Medline Search Strategy

Date of Search 11/12/2024

Search Update Period 4/01/2024–11/12/2024

Ovid MEDLINE(R) ALL <1946 to December 11. 2024>

1. exp Osteoarthritis/
2. osteoarthr$.tw.
3. (degenerative adj2 arthritis).tw.
4. arthrosis.tw.
5. or/1–4
6. exp knee/
7. exp Knee Joint/
8. knee$.tw.
9. or/6–8
10. exp exercise/
11. exercis*.tw.kf.
12. exp Exercise Therapy/
13. exp Exercise Movement Techniques/
14. exp Physical Therapy Modalities/
15. exp Recreation/
16. exp Physical Fitness/
17. pilates.tw.kf.
18. (Tai Chi or Tai Ji or Taiji or Taijiquan).tw.kf.
19. run$.tw.kf.
20. jog$.tw.kf.
21. treadmill$.tw.kf.
22. bicycl$.tw.kf.
23. (cycle$ or cycling).tw.kf.
24. walk$.tw.kf.
25. or/10–24
26. randomised controlled trial.pt.
27. controlled clinical trial.pt.
28. randomised.ab.
29. placebo.ab.
30. drug therapy.fs
31. randomly.ab.
32. trial.ab.
33. groups.ab.
34. or/19–26
35. exp animals/not humans.sh.
36. 34 not 35
37. 5 and 9 and 25 and 36
38. limit 37 to dt = 20240104–20241211

EMBASE Search strategy

Date of Search 11/12/2024

Search Update Period 04/01/2024–11/12/2024

Embase Classic + Embase <1946 to December 11. 2024>

1. exp osteoarthritis/
2. osteoarthr$.tw.
3. (degenerative adj2 arthritis).tw.
4. arthrosis.tw.
5. or/1–4
6. Knee/
7. knee$.tw.
8. or/6–7
9. exp EXERCISE/
10. exercis*.tw.kf.
11. exp fitness/
12. exp physiotherapy/
13. physical therapy.tw.kf.
14. exp pilates/
15. exp Tai Chi/
16. (Tai Chi or Tai Ji or Taiji or Taijiquan).tw.kf.
17. jog$.tw.kf.
18. treadmill$.tw.kf.
19. bicycl$.tw.kf.
20. (cycle$ or cycling).tw.kf.
21. walk$.tw.kf.
22. or/9–21
23. exp randomised controlled trial/
24. controlled clinical trial/
25. random$.ti.ab.
26. exp randomisation/
27. intermethod comparison/
28. placebo.ti.ab.
29. (compare or compared or comparison).ti.
30. ((evaluated or evaluate or evaluating or assessed or assess) and (compare or compared or comparing or comparison)).ab.
31. (open adj label).ti.ab.
32. ((double or single or doubly or singly) adj (blind or blinded or blindly)).ti.ab.
33. double blind procedure/
34. parallel group$1.ti.ab.
35. (crossover or cross over).ti.ab.
36. ((assign$ or match or matched or allocation) adj5 (alternate or group$1 or intervention$1 or patient$1 or subject$1 or participant$1)).ti.ab.
37. (assigned or allocated).ti.ab.
38. (controlled adj7 (study or design or trial)).ti.ab.
39. (volunteer or volunteers).ti.ab.
40. human experiment/
41. trial.ti.
42. or/23–41
43. 5 and 8 and 22 and 42
44. limit 43 to dc = 20240104–20241211

CENTRAL Search Strategy

Date of Search‐11/12/2024

Search Update Period 04/01/2024–11/12/2024

EBM Reviews—Cochrane Central Register of Controlled Trials <1946 to December 11. 2024>

1. exp Osteoarthritis/
2. osteoarthr$.tw.
3. (degenerative adj2 arthritis).tw.
4. arthrosis.tw.
5. or/1–4
6. exp knee/
7. exp Knee Joint/
8. knee$.tw.
9. or/6–8
10. exp exercise/
11. exercis*.tw.kf.
12. exp Exercise Therapy/
13. exp Exercise Movement Techniques/
14. exp Physical Therapy Modalities/
15. exp Recreation/
16. exp Physical Fitness/
17. pilates.tw.kf.
18. (Tai Chi or Tai Ji or Taiji or Taijiquan).tw.kf.
19. run$.tw.kf.
20. jog$.tw.kf.
21. treadmill$.tw.kf.
22. bicycl$.tw.kf.
23. (cycle$ or cycling).tw.kf.
24. walk$.tw.kf.
25. or/10–24
26. 5 and 9 and 25
27. limit 26 to yr = ‘2024’

## Appendix 2

Overview of the study characteristics for the 15 included studies.

**Table:**

Study (country)	Treatment groups	Sample size	Age (mean) years	Female (%)	Mean BMI (SD)	Osteoarthritis criteria	Length of intervention	Frequency per week	Volume (sets × repetitions × number of exercises)	Intensity	Total exercise dosage*	Number of consultations with a clinician
Baker et al. 2001 (United States of America)	Exercise	23	69.0 (6.0)	74	31.0 (4.0)	ACR criteria	16 weeks	3	168 (2 × 12 × 7)	Started at 3–5 on rating perceived exertion scale then progressed to 8	64,512	12
	Attention control	23	68.0 (6.0)	82	32.0 (5.0)							7
Bennell et al. 2010 (Australia)	Exercise	45	64.5 (9.1)	51	27.5 (4.7)	ACR criteria. KL grade II+. Medial tibiofemoral compartment disease	12 weeks	5	180 (3 × 10 × 6)	10 repetition max	75,600	60
	Wait list	44	64.6 (7.6)	46	28.4 (4.1)							0
Bruce‐Brand et al. 2012 (Ireland)	Exercise	14	63.4 (5.9)	60	33.9 (8.3)	KL grade III+	8 weeks	3	180 (3 × 10 × 6)	14 or above on rating perceived exertion	3780	18
	Standard care	13	65.2 (3.1)	50	31.7 (4.1)							Not reported
Chang et al. 2012, (Taiwan)	General physiotherapy + exercise	30	65.0 (8.4)	100	24.9 (3.3)	KL grade II or III. Knee flexion > 90°	8 weeks	2	30 (3 × 10 × 1)	13 on rating of perceived exertion	420	16
	General physiotherapy	30	70.8 (8.4)	100	25.7 (3.6)							16
de Almeida et al. 2020 (Brazil)	Exercise	22	55.2 (7.4)	76	26 (3.1)	ACR criteria. KL grade II or III	14 weeks	3	180 (2 × 15 × 6)	50% 1 rep max for quads and hamstrings; 25% 1 rep max for hip adductors and abductors	1620	42
	Attention control	22	53.8 (7.7)	80	27 (2.7)							6
Ettinger et al. 1997 (United States of America)	Exercise	144	68.0 (6.0)	73	Not reported	ACR criteria	12 weeks	3	120 (2 × 12 × 5)	2 × 12 repetitions max	1800	36
	Attention control	75	69.0 (6.0)	69	Not reported							3
Hsu et al. 2021 (Taiwan)	Diet + exercise	22	65.6 (3.9)	71	31.10 (2.6)	KL grade III+	12 weeks	3	250 (5 × 10 × 5)	13 rating perceived exertion	5250	24
	Diet	22	66.0 (3.9)	57	30.80 (2.6)							12
Imoto et al. 2012 (Brazil)	Exercise	50	61.5 (6.9)	90	29.7 (4.1)	ACR criteria	8 weeks	2	45 (3 × 15 × 1)	50%–60% 1 rep max	495	16
	Attention control	50	58.8 (9.6)	90	30.0 (5.1)							3
Jan et al. 2009 (Taiwan)	Exercise	71	62.6 (6.7)	79.4	Not reported	ACR criteria. KL grade < IV (most KL II)	8 weeks	3	24 (4 × 6 × 1)	50% 1 rep max. Increasing by 5% every 2 weeks over 8 weeks	432	24
	No intervention	35	62.2 (6.7)	69	Not reported							24
Karaborklu Argut et al. 2024 (Turkey)	Exercise platelet‐rich plasma	28	54 (7)	71	28 (4)	ACR criteria	6 weeks	2	150 (2 × 10 × 7.5)	10 repetition max	2100	12
	Platelet‐rich plasma	28	55 (7)	71	29 (4)							12
Lee et al. 2023 (Korea)	Exercise	18	65.6 (3.7)	100	24.3 (SD not reported)	KL grade II+	8 weeks	3	30 (2 × 15 × 1)	66% of max muscle strength	594	24
	Usual care	18	68.3 (4.8)	100	25.5 (SD not reported)							0
Pazit et al. 2018 (Australia)	Exercise	10	67.8 (6.2)	44	28.2 (5.6)	ACR criteria	8 weeks	2	77 (2.2 × 5 × 7)	Week 1–2 : 20%–40% 1RM Week 3–5: 40%–60% 1RM Week 6–8: 60%–80% 1RM	816	16
	Usual care	10	70.4 (7.8)	56	28.4 (3.9)							0
Samuel et al. 2014 (India)	Interferential + exercise	37	49.9 (SD not reported)	64.9	28.7 (SD not reported)	Chronic knee pain > 6 months and KL grade I‐III	4 weeks	5	50 (5 × 10 × 1)	50% 1 rep max. Progressing 5% each week	1500	20
	Interferentiaopl	36	50.8 (SD not reported)	69	26.8 (SD not reported)							10
Topp et al. 2002 (United States of America)	Exercise	67	63.3 (1.8)	71	Not reported	ACR criteria	16 weeks	3	132 (2 × 11 × 6)	Week 1 & 2: Perceived exertion rating of mild fatigue after 8 repetitions. Weeks 9–16: Perceived exertion rating of moderate fatigue after 12 repetitions	1188	48
	No intervention	35	60.9 (1.8)	80	Not reported							0

*Note:* Total exercise dosage is calculated using the following formula: Volume (sets × repetitions × number of exercises) × frequency per week × intensity × duration (length of the intervention).

## Appendix 3

Results of meta‐regressions for total exercise dosage without including covariates.

For change in pain: regression co‐efficient = < 0.01 (95% CI < −0.01 to < 0.01); *P* = 0.35. *I* ^2^ = 98%.

For change in function: regression co‐efficient = < 0.01 (95% CI < −0.01 to < 0.01); *P* = 0.11. *I* ^2^ = 79%.

## Appendix 4

Results of the meta‐regression for individual dosage variables and changes in pain (including covariates (1) type of comparator, and (2) the total number of consultations with a clinician throughout the intervention).

**Table:**

	Regression co‐efficient (0–100 scale)	95% confidence interval	*p*‐value	*I* ^2^
Frequency	− 3.81	− 17.55 to 9.93	0.54	98%
Volume	0.09	− 0.05 to 0.25	0.18	97%
Intensity	4.06	− 4.08 to 12.21	0.28	97%
Duration	1.85	− 1.98 to 5.70	0.30	97%

Results of the meta‐regression for individual dosage variables and changes in function (including covariates (1) type of comparator, and (2) the total number of consultations with a clinician throughout the intervention).

**Table:**

	Regression co‐efficient (on a 0–100 scale)	95% confidence interval	*p*‐value	*I* ^2^
Frequency	− 2.20	− 10.09 to 5.68	0.54	78%
Volume	0.09	< 0.01 to 0.15	0.05	65%
Intensity	2.10	− 2.74 to 6.92	0.35	77%
Duration	0.91	− 1.41 to 3.22	0.39	77%

## Appendix 5

Funnel plots and Egger's tests investigating publication bias for pain and function.

Egger's test for pain: Intercept = − 4.42; 95% CI: − 34.07 to 25.22; *p* = 0.75.

Egger's test for function: Intercept = − 4.27; 95% CI: − 32.92 to 24.37; *p* = 0.75.

## Appendix 6

Results of meta‐regressions for volume and changes in function.
